# Learning Stiffness Tensors in Self‐Activated Solids via a Local Rule

**DOI:** 10.1002/advs.202308584

**Published:** 2024-03-14

**Authors:** Yuxuan Tang, Wenjing Ye, Jingjing Jia, Yangyang Chen

**Affiliations:** ^1^ Department of Mechanical and Aerospace Engineering The Hong Kong University of Science and Technology Clear Water Bay Kowloon Hong Kong; ^2^ Institute of Materials Engineering Beijing Institute of Collaborative Innovation Beijing 100094 China

**Keywords:** active metamaterials, adaptive stiffness, lattice metamaterials

## Abstract

Mechanical metamaterials are often designed with particular properties for specific load‐bearing functions. Alternatively, this study aims to create a class of active lattice metamaterials, dubbed self‐activated solids, that can learn desired stiffness tensors from the elastic deformations they experienced, a crucial feature to improve the performance, efficiency, and functionality of materials. Artificial adaptive matters that combine sensory, control, and actuation elements can offer appealing solutions. However, challenges still remain: The designs will rely on accurate off‐line and global computations, as well as intricate coordination among individual elements. Here, a simple online and local learning strategy is initiated based on contrastive Hebbian learning to gradually guide self‐activated solids to possess sought‐after stiffness tensors autonomously and reversibly. During learning, the bond stiffness of the active lattice varies depending only on its local strain. The numerical tests show that the self‐activated solid can not only achieve the desired bulk, shear, and coupling moduli but also manifest uni‐mode and bi‐mode extremal materials by itself after experiencing the corresponding elastic deformations. Further, the self‐activated solid can also achieve the desired time‐varying moduli when exposed to temporally different loads. The design is applicable to any lattice geometries and is resistant to damage and instabilities. The material design approach and the physical learning strategy suggested can benefit the design of autonomous materials, physical learning machines, and adaptive robots.

## Graphical abstract

In this article, a methodology is suggested to design a class of active lattice metamaterials that can physically and autonomously learn and adjust their stiffness tensors at will in response to external loads. The design is scalable and applicable to other discrete materials. The methodology can benefit the design of autonomous materials, physical learning machines, and adaptive robots.

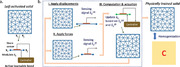

## 1 Introduction

Materials and structures are usually designed to meet predefined properties or functions, which nonetheless are often invariant to external loading conditions.^[^ ^1^, ^2^, ^3^, ^4^, ^5^, ^6^, ^7^, ^8^ ^]^ As a matter of fact, solid materials capable of adapting their mechanical properties in response to load changes can surprisingly improve their performance, functionality, versatility, and sustainability and benefit applications ranging from aerospace and automotive engineering to soft robotics, biomedical and consumer products.^[^ ^9^, ^10^, ^11^, ^12^, ^13^, ^14^, ^15^, ^16^, ^17^, ^18^, ^19^, ^20^ ^]^ Passive materials or systems that respond differently to different types of input loads (usually low or high amplitude vibration or shock loads) include delicately designed bulked and viscoelastic materials, where a small number of different responses can be achieved using geometric designs.^[^ ^21^, ^22^, ^23^ ^]^ On the other hand, advances in stimuli‐responsive materials integrated with sophisticated sensors and electrical control systems allow the construction of engineered adaptive matters.^[^ ^24^, ^25^, ^26^, ^27^, ^28^, ^29^, ^30^, ^31^ ^]^ Those adaptive matters have been successfully demonstrated in the form of active and robotic metamaterials for realizing tunable and odd mechanical properties,^[^ ^32^, ^33^, ^34^, ^35^, ^36^, ^37^, ^38^ ^]^ controlling mechanical vibrations and waves,^[^ ^32^, ^33^, ^35^, ^36^, ^39^, ^40^, ^41^ ^]^ and serving as a compelling platform to exploit phenomena in non‐Hermitian mechanics.^[^ ^36^, ^37^, ^38^, ^40^, ^42^, ^43^, ^44^, ^45^ ^]^ However, designing materials with load‐adaptive stiffness using optimization methods would rely on off‐line and global computations and require complex coordination among external sensory, global control, and internal actuation elements, making them vulnerable to damages and instabilities. Further, the loads that the materials are expected to undergo should always be given in advance. Unexpected loads are unable to be considered in the design, limiting the application scope of engineered adaptive matters.

Here, inspired by contrastive Hebbian learning, we suggest a simple online and local learning strategy leading to materials that can learn desired stiffness tensors based on elastic deformations they experienced. Contrastive Hebbian learning is a type of supervised learning algorithm to learn feature representations by comparing two different inputs in a contrastive manner.^[^ ^46^, ^47^, ^48^ ^]^ Recently, employing potential energies of the two states of the system as the two different inputs, contrastive Hebbian learning has inspired the development of completely distributed and physics‐driven learning machines.^[^ ^49^, ^50^, ^51^, ^52^, ^53^ ^]^ In those designs, learning degrees of freedom are updated based on only local conditions. Consequently, the method can be more scalable than artificial neural networks and the system is more resistant to damage. Using this approach, physical learning machines have been successfully demonstrated in an electrical network experimentally^[^ ^54^ ^]^ and flow and elastic networks theoretically.^[^ ^49^ ^]^ These physical learning machines are responsible for structure functions and, at the same time, perform the task of adapting those structure functions according to environmental changes. Thus, contrastive Hebbian learning can be an appealing online, and local, strategy that solid materials are able to implement to adapt their mechanical properties in response to load changes to enhance their performance and functionality. However, it is unknown how contrastive Hebbian learning can be implemented to regulate the stiffness tensors of solid materials.

**Figure 1:**
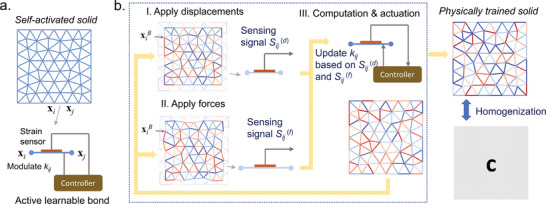
Self‐activated solid and local learning rule. a) The self‐activated solid consists of a set of active learnable bonds interconnected in a 2D space. The active bond contains a strain sensor, a controller, and an actuating element that modulates the stiffness of the bond. b) The local learning rule embodies a series of learning iterations. Each of the iterations includes three steps: I) Apply displacement boundary conditions to realize a homogenized strain state and measure the local strain of each bond; II) Apply force boundary conditions to realize a homogenized stress state and measure the local strain of each bond; III) Update the stiffness of each bond using local measurements. The self‐activated solid displays the desired stiffness tensor after physical training.

In this work, we suggest creating a class of active lattice metamaterials, called self‐activated solids, and define the solid's two states as elastic deformations induced by displacement and force boundary conditions for the implementation of contrastive Hebbian learning (see **Figure** **1**). A local learning rule is derived to achieve the desired stiffness tensors through repeated elastic deformations, where the bond stiffness (the learning degrees of freedom) is required to be updated based on the local strain of each bond in the lattice (see Figure 1b). A range of numerical simulations is performed to validate the design principle and demonstrate that the self‐activated solid can be physically trained with elastic deformations to display the desired bulk, shear, and coupling moduli. The solids can also behave as uni‐mode and bi‐mode extremal materials themselves after the corresponding training. Finally, time‐varying moduli are realized with temporally different loads. The design is scalable and applicable to other discrete materials with arbitrary geometries. The design framework of the physical learning material we initiate can lead to functional and efficient autonomous materials, machines, and robots.

## 2 Results and Discussion

### 2.1 Self‐Activated Solids

The self‐activated solid consists of a set of active learnable bonds interconnected in a 2D space (see Figure 1a). The two nodes of the active bond (*i*, *j*) are positioned at **x**_*i*_ and **x**_*j*_, and the stiffness of the linear bond is *k*_*ij*_. If the bond between **x**_*i*_ and **x**_*j*_ does not exist, *k*_*ij*_ = 0. The displacement of the self‐activated solid at **x**_*i*_ is denoted by **U**_*i*_. The active bond is constructed by attaching a strain sensor to the bond and connecting the sensor to a controller, which then generates an output signal to modulate the stiffness of the bond *k*_*ij*_ following a local learning rule proposed in this study. To modulate the stiffness, the active bond can be designed using stimuli‐responsive materials that change their material properties under different electrical, magnetic, or thermal fields,^[^ ^9^, ^19^ ^]^ or by implementing electrical feedback loops over materials having electromechanical couplings.^[^ ^16^, ^24^, ^55^ ^]^ We also suggest a design composed of a spring, a shaft, and a step motor‐powered rotator for experimental realizations (see Supporting Information for details). Note that the suggested approach would be a simple platform to demonstrate behavior, and the working principles are generalizable to many different types of actuation and energization.

To describe the elastic properties of the self‐activated solid, we introduce an effective stiffness tensor defined by

(1) $$\begin{array}{c} \left[\begin{array}{c} \Sigma_{1}\\ \Sigma_{2}\\ \Sigma_{3} \end{array}\right]=\left[\begin{array}{ccc} B & c_{1} & c_{2}\\ c_{1} & \mu_{1} & c_{3}\\ c_{2} & c_{3} & \mu_{2} \end{array}\right]\left[\begin{array}{c} E_{1}\\ E_{2}\\ E_{3} \end{array}\right] \end{array}$$

where the homogenized stresses Σ_1_ = σ_11_ + σ_22_, Σ_2_ = σ_11_ − σ_22_, and Σ_3_ = σ_12_ + σ_21_ denoting the hydrostatic pressure, shear stress I, and shear stress II, respectively, in which σ_*ij*_ are the homogenized Cauchy stress components. Homogenized strains are defined in a similar way as *E*_1_ = *u*_1, 1_ + *u*_2, 2_, *E*_2_ = *u*_1, 1_ − *u*_2, 2_, and *E*_3_ = *u*_1, 2_ + *u*_2, 1_ representing the hydrostatic strain, shear strain I, and shear strain II, respectively, in which *u*_*i*_ is the displacement. Consequently, *B*, μ_1_, and μ_2_ are homogenized bulk, shear I, and shear II modulus, respectively, and *c*_1_, *c*_2_, and *c*_3_ are the corresponding coupling moduli. Note that here we ignore the body torque Σ_4_ = σ_12_ − σ_21_ and the rigid‐body rotation *E*_4_ = *u*_1, 2_ − *u*_2, 1_, thanks to the fact that the self‐activated solid is free‐standing and does not have connections to the reference ground.

### 2.2 Local Learning Rule

To supervise the self‐activated solid to achieve the desired load‐adaptive stiffness tensor, we derive a local learning rule based on contrastive Hebbian learning. We focus on the desired bulk modulus first, and then gradually extend the learning rule to other components of the stiffness tensor. To physically train the self‐activated solid to attain the desired bulk modulus *B* ^(0)^, we first impose a set of displacement boundary conditions at boundary nodes $x_{i}^{B}$ of the self‐activated solid to produce an effective strain $E_{1}^{\left(0\right)}$, where the imposed displacement $U_{i}^{B}=e_{0}x_{i}^{B}$ with **e**_0_ being the corresponding Cauchy strain tensor of the strain state ${\left[\begin{array}{ccc} E_{1}^{\left(0\right)} & 0 & 0 \end{array}\right]}^{T}$ (see the upper left panel in Figure 1b). In this displacement‐prescribed state, each of the active bonds deforms and generates a sensing signal $S_{ij}^{\left(d\right)}$ denoting the change in length of the spring (*i*, *j*). Reaction forces $f_{i}^{B}$ also emerge at $x_{i}^{B}$ to sustain the deformation. Clearly, if the effective bulk modulus of the self‐activated solid is not equal to *B* ^(0)^, the reaction forces produced cannot induce an effective stress state described by ${\left[\begin{array}{ccc} \Sigma_{1}^{\left(0\right)} & 0 & 0 \end{array}\right]}^{T}$, where $\Sigma_{1}^{\left(0\right)}=B^{\left(0\right)}E_{1}^{\left(0\right)}$. Next, we apply a set of force boundary conditions to actively induce an effective stress state ${\left[\begin{array}{ccc} \Sigma_{1}^{\left(0\right)} & 0 & 0 \end{array}\right]}^{T}$ over the self‐activated solid, where the force at $x_{i}^{B}$ is denoted by $F_{i}^{B}=t_{0}s\left(x_{i}^{B}\right)A\left(x_{i}^{B}\right)/n\left(x_{i}^{B}\right)$ with **t**_0_, $s\left(x_{i}^{B}\right)$, $A\left(x_{i}^{B}\right)$, and $n\left(x_{i}^{B}\right)$ being the corresponding Cauchy stress tensor of the stress state ${\left[\begin{array}{ccc} \Sigma_{1}^{\left(0\right)} & 0 & 0 \end{array}\right]}^{T}$, the unit normal vector, the area, and the total number of nodes of the boundary where $x_{i}^{B}$ occupies, respectively. (see the lower left panel in Figure 1b). Similarly, in this force‐prescribed state, each of the active bonds deforms and generates a sensing signal $S_{ij}^{\left(f\right)}$. The induced effective strain state is also not equal to ${\left[\begin{array}{ccc} E_{1}^{\left(0\right)} & 0 & 0 \end{array}\right]}^{T}$ when the effective bulk modulus of the self‐activated solid is not equal to *B* ^(0)^. In the third step, the linear stiffness *k*_*ij*_ of each of the active bonds is updated based on the sensing signals $S_{ij}^{\left(d\right)}$ and $S_{ij}^{\left(f\right)}$. To update *k*_*ij*_, our objective is to let the difference between the elastic strain energies of the displacement‐ and force‐prescribed states gradually decrease and infinitely approach zero. Once the strain energies of the two states are equal, the work done due to $f_{i}^{B}$ and $F_{i}^{B}$ should be the same, and the self‐activated solid displays the desired effective bulk modulus *B* ^(0)^ automatically. We then divide the task by requiring the elastic energies of each of the individual bonds at the two states to be gradually equal after updating its stiffness. To find the updating rule of the individual bond, we consider the fact that, i.e., for a self‐activated solid composed of just one active bond, when the bond is ”stiffer” (or ”softer”) than the desired value, the elastic energy of the force‐prescribed state is lower (or higher) than the elastic energy of the displacement‐prescribed state. The fact indicates that when $\frac{1}{2}k_{ij}{\left[S_{ij}^{\left(f\right)}\right]}^{2}<\frac{1}{2}k_{ij}{\left[S_{ij}^{\left(d\right)}\right]}^{2}$ (or $\frac{1}{2}k_{ij}{\left[S_{ij}^{\left(f\right)}\right]}^{2}>\frac{1}{2}k_{ij}{\left[S_{ij}^{\left(d\right)}\right]}^{2}$), *k*_*ij*_ needs to decrease (or increase) to reduce the difference between the two states. Thus, we extend this fact to the self‐activated elastic network and suggest a learning rule, which reads

(2) $$\begin{array}{c} \begin{array}{c} \Delta k_{ij}\propto \left\{\frac{1}{2}k_{ij}{\left[S_{ij}^{\left(f\right)}\right]}^{2}-\frac{1}{2}k_{ij}{\left[S_{ij}^{\left(d\right)}\right]}^{2}\right\} \end{array} \end{array}$$

where Δ*k*_*ij*_ denotes the change in linear stiffness of the active bond. Equation (2) can be simplified by

(3) $$\begin{array}{c} \begin{array}{c} \Delta k_{ij}=\gamma \left\{{\left[S_{ij}^{\left(f\right)}\right]}^{2}-{\left[S_{ij}^{\left(d\right)}\right]}^{2}\right\} \end{array} \end{array}$$

where γ represents the learning rate. Examining Equation (3), we have three important observations. First, the learning rule is entirely local in the sense that the change in the stiffness of the spring (*i*, *j*) depends only on its local strains in the two different deformation states, $S_{ij}^{\left(f\right)}$ and $S_{ij}^{\left(d\right)}$, offering appealing advantages for the implementation of active materials and machines. Second, when the iterations are convergent, the strain energies of the displacement‐ and force‐prescribed states are equal, as ${\left[S_{ij}^{\left(f\right)}\right]}^{2}={\left[S_{ij}^{\left(d\right)}\right]}^{2}$. The self‐activated solid displays the desired modulus. Lastly, the local learning rule is independent of the signs of applied displacements and forces, since Δ*k*_*ij*_ is a function of ${\left[S_{ij}^{\left(f\right)}\right]}^{2}$ and ${\left[S_{ij}^{\left(d\right)}\right]}^{2}$. Thus, during physical training, the deformation can be tension or compression. It should be noted that the local learning rule suggested in Equation (3) does not need to determine the displacements and forces at the boundary nodes in each of the iterations. These displacements and forces during the entire training are constant and predetermined, in stark contrast to equilibrium propagation and coupled learning, where a global supervisor is required to measure the responses at the target nodes and determine the new targets in each of the iterations.^[^ ^49^ ^]^ As a result, the local learning rule in Equation (3) can be more convenient and easier for experimental realizations. Note also that we neglect the strain energy of the elastic lattice from other contributions, i.e., rotational interactions between each of the bonds. These effects can be leveraged by extending Equation (3) to include more learning degrees of freedom for the training.

### 2.3 Learning Desired Bulk Modulus

**Figure 2:**
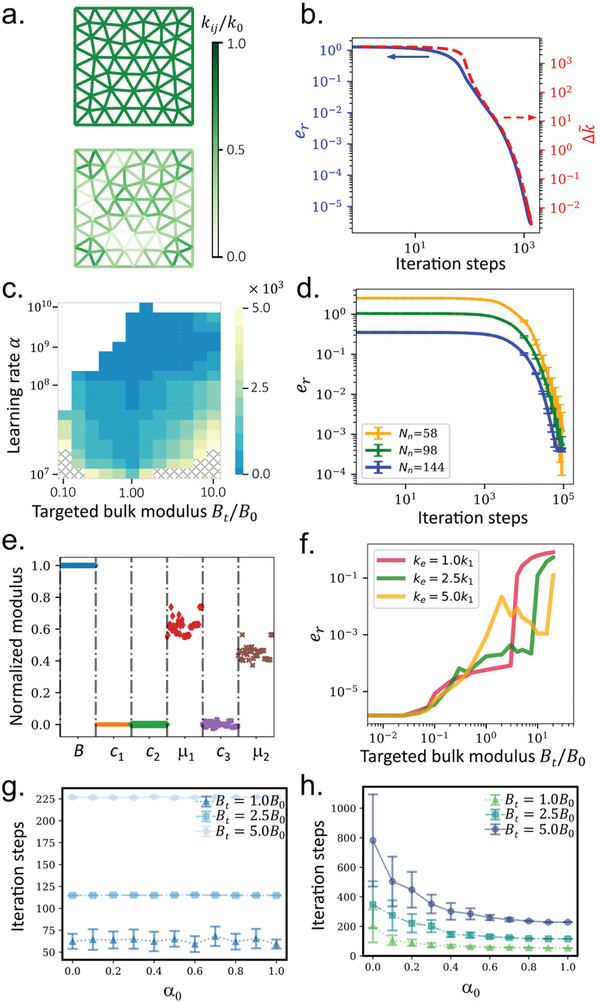
The self‐activated solid is trained with a desired bulk modulus. a) Geometry and bond stiffness of the self‐activated lattice before and after training. b) Evolution of the force error and change in bond stiffness of the self‐activated lattice during training. c) Numbers of learning steps needed for convergence with different target bulk moduli and learning rates. d) Evolution of the force error of the self‐activated lattice with different numbers of nodes during learning. e) Normalized effective bulk moduli of the trained self‐activated solid with different initial bond stiffness. f) Final force error of the trained self‐activated solid with different target bulk moduli, where different upper bounds on the bond stiffness are implemented. g,h) Number of iteration steps needed for convergence when imposing different α_0_ over the initial stiffness (g) and learning rate (h) of the active bonds.

Having derived the local learning rule, we now implement it into a self‐activated solid to numerically test its performance. The geometry of the self‐activated solid is built using Pygmsh, a mesh generating tool with the versatility of Python. A triangular lattice occupied in a square (with side length *L* = 1 m) is generated using the Delaunay triangulation algorithm^[^ ^56^ ^]^ (see the top panel in **Figure** **2**a). We set the mesh size as *L*/*N*_*e*_, where *N*_*e*_ denotes the number of bonds on the edge of the rectangle. In the figure, *N*_*e*_ = 7 and the total number of nodes *N* is 58. The initial stiffness of all active bonds is the same with *k*_*ij*_ = *k*_0_ = 3 × 10^4^ Nm^‐1^. The bond stiffness is enforced to be non‐negative all the time. In the first example of physical training, we select *B* ^(0)^ = 5 × 10^4^ Nm^−2^, $E_{1}^{\left(0\right)}=0.01$, and $\Sigma_{1}^{\left(0\right)}=500$ Nm^−2^. The other stress and strain components are equal to zero. During training, the same sets of displacement and force boundary conditions are repeatedly applied with the normalized learning rate $\gamma_{0}=\frac{\gamma L}{B^{\left(0\right)}}=10^{3}$. After training, the self‐activated solid learns the desired bulk modulus *B* ^(0)^, where all the bond stiffness decreases to desired values (see the bottom panel in Figure 2a). Figure 2b shows the evolution of the bond stiffness and the error during the whole training process. Here, we define the total change in stiffness $\Delta \tilde{k}=\sqrt{\sum_{i,j}\Delta k_{ij}^{2}}$ and the relative force error $e_{r}=\sqrt{\sum_{i}{∥F_{i}^{\left(d\right)}-F_{i}^{\left(f\right)}∥}^{2}}/\sqrt{\sum_{j}{∥F_{j}^{\left(f\right)}∥}^{2}}$, where $F_{i}^{\left(d\right)}$ and $F_{i}^{\left(f\right)}$ are the reaction force in the displacement‐prescribed state and the applied force in the force‐prescribed state at the *i*‐th node, respectively. It can be seen from Figure 2b that both the force error (solid blue curve) and the total change in stiffness (dashed red curve) decrease to small values as the iteration approaches the 10^3^‐th steps, indicating the success of the local learning rule suggested in the study.

As advised by Equation (3), the learning rate γ_0_ is an important parameter that determines not only the number of iterations to convergence, but the success of the learning process. To find the optimal learning rate for different learning objectives and to know its limitations, we perform a series of studies by changing the target bulk modulus *B*_*t*_ from 0.1*B*_0_ to 10.0*B*_0_ (*B*_0_ = 10^5^ Nm^−2^) with the normalized learning rate γ_0_ ranging from 10^2^ to 10^5^ (see Figure 2c). In the study, the initial bond stiffness remains unchanged as in Figure 2a,b. In Figure 2c, when the error is less than 10^−2^, we consider the result convergent and the learning success. We also set a maximum number of iterations in the calculations as 5 × 10^3^. The color in the figure represents the number of iterations to convergence. The white and shaded areas denote the regions where no convergence has been found during the entire calculation. Clearly, hundreds of iterations could be enough for most of the cases when the learning rate is properly selected from the blue region. Increasing the targeted bulk modulus *B*_*t*_ generally requires larger values of the optimal learning rate. Further, decreasing the learning rate will increase the number of iterations to convergence. For some cases, even 5 × 10^3^ iterations are not enough (shaded regions). On the other hand, excessively large learning rates will let the system oscillate without convergence (white regions). Learning rates should be selected so that ${\left[S_{ij}^{\left(f\right)}\right]}^{2}-{\left[S_{ij}^{\left(d\right)}\right]}^{2}$ does not constantly change sign. This way the difference in strain energy between the two states can always be reduced. It may also be necessary to mention that the initial bond stiffness has an impact on the optimal learning rate, which should be considered during the learning process.

The local learning rule developed in this study is applicable to any lattice geometries. To show this, we regenerate a range of lattices containing different numbers of nodes and perform the physical learning tasks over them, where the other parameters are left unchanged. Figure 2d shows the evolution of the error, when the number of nodes *N* is equal to 58, 98, and 144. In the figure, ten different lattices are generated for each of the three curves. We can clearly see that all the physical learning tasks are successful with sufficiently small errors at the end of the tasks, even if the initial errors are different among those lattices.

Having examined the effects of geometry, we now investigate the consequences of different initial bond stiffness. In Figure 2e, we calculate the effective stiffness tensors of the trained self‐activated solids with different initial bond stiffness (see Supporting Information for the detailed calculation method). The moduli in the figure are rescaled by *B*_0_, and the initial stiffness of the bond is randomized by *k*_*ij*_ = *k*_0_ · *U*[0, 1], where *U* is the uniform distribution function. As expected, all solids display the desired bulk modulus after training. Note that this training also gives rise to an unintended effect in which the coupling moduli *c*_1_ and *c*_2_ are required to be zero. This can be easily checked by substituting stress components ${\left[\begin{array}{ccc} \Sigma_{1}^{\left(0\right)} & 0 & 0 \end{array}\right]}^{T}$ and strain components ${\left[\begin{array}{ccc} E_{1}^{\left(0\right)} & 0 & 0 \end{array}\right]}^{T}$ into Equation (1) to solve the moduli. By doing so, we also note that the other three moduli are left out of control during training such that μ_1_, μ_2_, and *c*_3_ are randomly distributed in Figure 2e, albeit within some ranges. The results in Figure 2e also illustrate that the solutions of *k*_*ij*_ are not unique for a desired modulus, offering possibilities to control the full stiffness tensor using the local learning rule.

Considering experimental implementation, it is practical to enforce maximum bond stiffness in the local learning of the self‐activated solid. Next, we study this factor and identify the maximum achievable bulk modulus with this enforced constraint. Figure 2f shows the error in the final step of training with different target bulk modulus *B*_*t*_ for different maximum values of bond stiffness *k*_*e*_. In the figure, the constant *k*_1_ = 10^5^ Nm^‐1^. When the target bulk modulus is small, the final error is quite low, indicating the success of the training. When the target bulk modulus passes through a critical value, the final error jumps and the self‐activated solid no longer satisfies the target bulk modulus. For the current setup, we find a linear relationship between the maximum bond stiffness and the critical value of the target bulk modulus with a factor roughly equal to 15 m^−1^.

Next, we focus on the variation effect of the parameters of the active bonds. We first enforce a uniform distribution function *P* = *U*[α_0_, 2 − α_0_] to the initial bond stiffness (*k*_*ij*_ = *k*_0_ *P*), where 0 ⩽ α_0_ ⩽ 1. In the study, all the other parameters are the same as those used in Figure 2e. Figure 2g shows the number of iteration steps needed to complete the learning of a desired bulk modulus when implementing different α_0_. For each of the points in the figure, we create ten different self‐activated solids using the same α_0_ for the training. The number of iteration steps for convergence is selected when the relative error is reduced to or less than 10^−4^. During the training, we impose the maximum number of iteration steps as 5 × 10^3^. As shown in the figure, the number of iteration steps for convergence is almost unchanged with different α_0_ for different desired bulk modulii. This is understandable as the relative error and the change in stiffness are reduced exponentially as the iteration proceeds (see Figure 2b), so as the variation in initial stiffness would not produce visible effects on the number of iteration steps for convergence. We then examine the variation effect of the learning rate by enforcing the same uniform distribution function *P* on the learning rate of the active bonds. Figure 2h shows the number of iteration steps needed for convergence when implementing different α_0_. It can be seen from the figure that when α_0_ is greater than 0.5, the effect of variation in the learning rate is nearly unnoticeable. All those self‐activated solids need almost the same number of iteration steps for convergence. However, when α_0_ is less than 0.5, the average number of iteration steps for convergence increases as α_0_ decreases, and, at the same time, those self‐activated solids behave quite differently from each other as evidenced by the increased standard deviation of the number of iteration steps. Since the learning rate controls the slope of exponential decrease in error, the quite small learning rate in the active bonds that requires large stiffness changes could need a much larger number of iteration steps for convergence. Consequently, when α_0_ is small, both the average number of iteration steps and its standard deviation become large. The results and conclusions demonstrated in Figure 2 for the bulk modulus are also valid for shear moduli μ_1_ and μ_2_ (see Supporting Information for details).

### 2.4 Learning Desired Coupling Moduli

**Figure 3:**
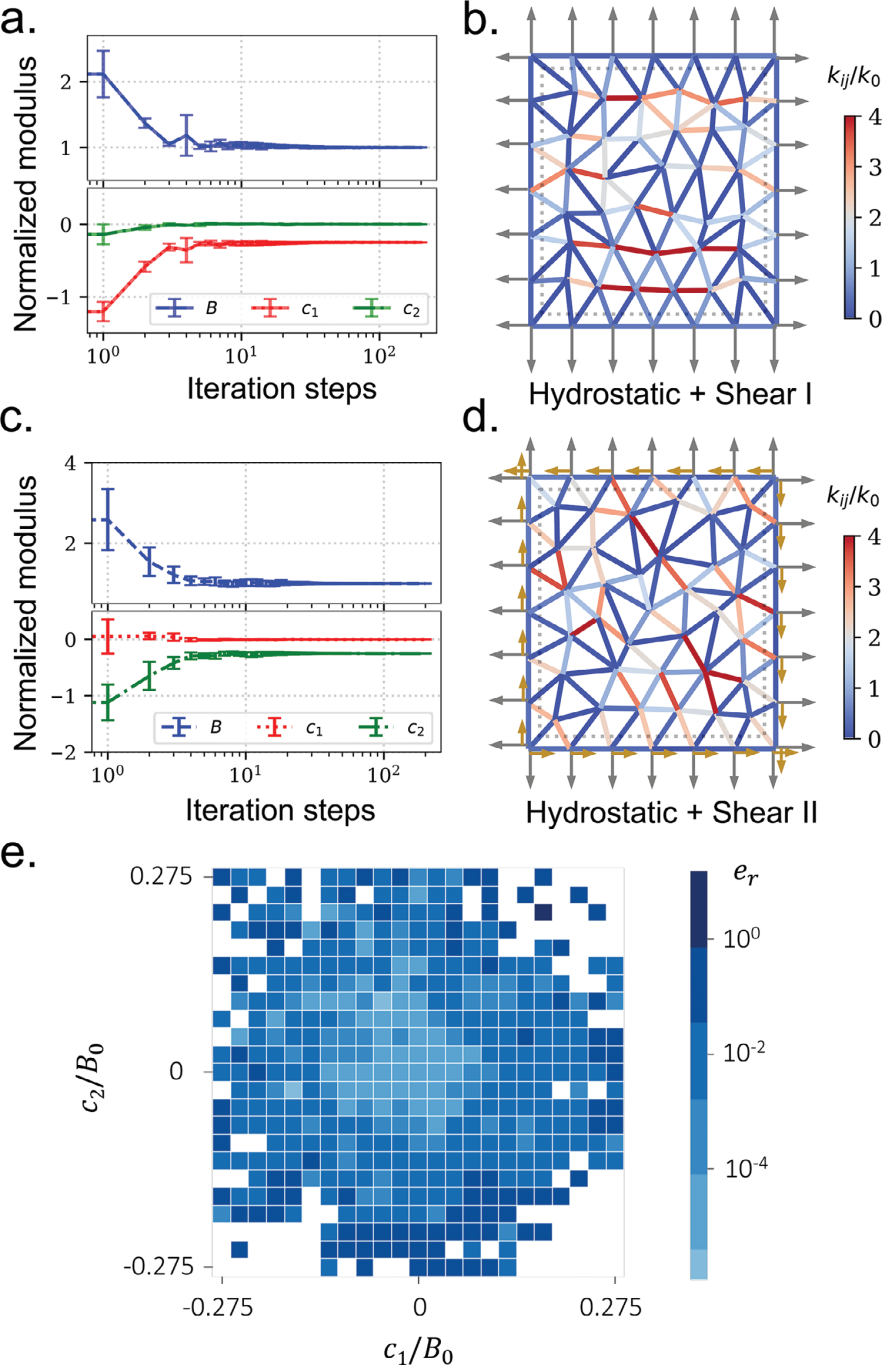
The self‐activated solid is trained with desired coupling moduli. a) Evolution of the normalized moduli of the self‐activated solid during learning, where we impose the target moduli *B* = *B*_0_, *c*_1_ = −0.25*B*_0_, and *c*_2_ = 0. b) Bond stiffness of a trained self‐activated solid. The arrows in the figure denote the hydrostatic and shear I stresses induced by a hydrostatic strain. c) Evolution of the normalized moduli of the self‐activated solid during learning, where we impose the target moduli *B* = *B*_0_, *c*_1_ = 0, and *c*_2_ = −0.25*B*_0_. d) Bond stiffness of a trained self‐activated solid. The arrows in the figure denote the hydrostatic and shear II stresses induced by a hydrostatic strain. e) Averaged force error of the training with different coupling moduli. During the training, the normalized learning rate γ_0_ = 10^3^, *N*_*e*_ = 7, and the initial bond stiffness *k*_*ij*_ = 10^4^ Nm^‐1^.

As indicated in Figure 2e, the training process for the bulk modulus also imposes strict control over the coupling moduli *c*_1_ and *c*_2_. Different from Figure 2e where *c*_1_ = *c*_2_ = 0, here we exploit physical learning to achieve desired nonzero coupling moduli to unlock the complex coupling between those stress and strain components, for example, the hydrostatic strain induces not just the hydrostatic stress but shear I and/or shear II stresses. In **Figure** **3**a, we employ the same strain components ${\left[\begin{array}{ccc} E_{1}^{\left(0\right)} & 0 & 0 \end{array}\right]}^{T}$ for the displacement‐prescribed state but different stress components ${\left[\begin{array}{ccc} \Sigma_{1}^{\left(0\right)} & \Sigma_{2}^{\left(0\right)} & 0 \end{array}\right]}^{T}$ for the force‐prescribed state, where $\Sigma_{2}^{\left(0\right)}=-0.25B_{0}E_{1}^{\left(0\right)}$. Consequently, the target moduli become *B* = *B*_0_, *c*_1_ = −0.25*B*_0_, and *c*_2_ = 0. In the study, we perform ten tests with different initial bond stiffnesses. The normalized moduli retrieved during simulations show that the desired moduli can be trained in a few hundred iterations for all tests (see Figure 3a). Figure 3b illustrates one of the trained self‐activated solids, where the color represents the bond stiffness after training. It can be clearly seen that when the solid experiences a hydrostatic strain, both the hydrostatic and shear I stresses are induced (see the force arrows in Figure 3b). Similarly, in Figure 3c, we train a self‐activated solid by selecting the target moduli as *B* = *B*_0_, *c*_1_ = 0, and *c*_2_ = −0.25*B*_0_, where the strain and stress components are ${\left[\begin{array}{ccc} E_{1}^{\left(0\right)} & 0 & 0 \end{array}\right]}^{T}$ and ${\left[\begin{array}{ccc} \Sigma_{1}^{\left(0\right)} & 0 & \Sigma_{3}^{\left(0\right)} \end{array}\right]}^{T}$, respectively, in which $\Sigma_{3}^{\left(0\right)}=-0.25B_{0}E_{1}^{\left(0\right)}$. As expected, when we apply a hydrostatic strain to the solid, both the hydrostatic and shear II stresses are induced (see the force arrows in Figure 3d). To check the validity of controlling *c*_1_ and *c*_2_ simultaneously, we perform a series of learning tasks by prescribing different desired values of *c*_1_ and *c*_2_ in Figure 3e. In each of the tasks, the learning stops at the 5 × 10^4^‐th iteration step. We calculate the error by averaging the force error *e*_*r*_ over the last one hundred steps. In the figure, the white areas denote the learning tasks that are unsuccessful due to failure of convergence. From the figure, we can safely conclude that, as long as $\sqrt{c_{1}^{2}+c_{2}^{2}}\leq 0.25B_{0}$, the current geometric setup is able to generate satisfactory learning outcomes. When $\sqrt{c_{1}^{2}+c_{2}^{2}}>0.25B_{0}$, we may need to change the geometry of the lattice, e.g., the type of mesh, chiral lattices, to incorporate geometric effects with the learning rule.

### 2.5 Learning to Behave as Extremal Materials

It has been shown that the local learning rule performs exceptionally well in the training of self‐activated solids to achieve some desired moduli, such as *B*, *c*_1_, and *c*_2_, simultaneously. However, it should be noted that the way of training we selected implements the same sets of displacement and force boundary conditions repeatedly applied over the entire iterations. This implementation leads to constraints on target moduli that we are able to prescribe during training. To see the constraints, we again substitute the implemented stress and strain components, ${\left[\begin{array}{ccc} \Sigma_{1}^{\left(0\right)} & \Sigma_{2}^{\left(0\right)} & \Sigma_{3}^{\left(0\right)} \end{array}\right]}^{T}$ and ${\left[\begin{array}{ccc} E_{1}^{\left(0\right)} & E_{2}^{\left(0\right)} & E_{3}^{\left(0\right)} \end{array}\right]}^{T}$, into Equation (1). Consequently, only three linear equations are attained, which nevertheless are needed to determine the six independent moduli, making it impossible to obtain unique solutions. One of the natural ways to have unique solutions for parts of the moduli is to select the strain with only one nonzero component. In this way, three moduli in one column of the stiffness tensor can be learned individually, as demonstrated in previous sections. Next, to train the six moduli in the stiffness tensor individually and simultaneously, we introduce another learning scheme with mixed sets of displacement and force boundary conditions applied over iterations.

Our specific focus in this section is on extremal materials, or called degenerate materials, which have one or multiple zero (or soft) modes ‐ elastic energy is equal to zero under one or multiple particular deformations.^[^ ^57^ ^]^ The first example we illustrate is to train a self‐activated solid to be a uni‐mode material, where we let shear II deformations produce zero stress, and all the other deformations induce nonzero stresses. Based on this property, we can easily find a target stiffness tensor satisfying all requirements, which reads

(4) $$\begin{array}{c} \begin{array}{c} c_{t}=\left[\begin{array}{ccc} B_{0} & 0 & 0\\ 0 & B_{0} & 0\\ 0 & 0 & 0 \end{array}\right] \end{array} \end{array}$$

To train this full stiffness tensor, we first generate two datasets composed of different sets of displacement and force boundary conditions for the soft and rigid modes, respectively. In particular, the rigid mode dataset contains different displacement and force boundary conditions corresponding to linear combinations of two orthogonal rigid strain states ${\left[\begin{array}{ccc} E_{1}^{\left(0\right)} & 0 & 0 \end{array}\right]}^{T}$ and ${\left[\begin{array}{ccc} 0 & E_{2}^{\left(0\right)} & 0 \end{array}\right]}^{T}$ and a stress state calculated using Equation (1) for the combined strain state, respectively. The soft mode dataset contains one set of displacement and force boundary conditions corresponding to a soft strain state ${\left[\begin{array}{ccc} 0 & 0 & E_{3}^{\left(0\right)} \end{array}\right]}^{T}$ and a zero stress state, respectively. To balance the dateset of the rigid modes so that the soft and rigid modes can be trained equally, we repeat this set of data multiple times so that the number of datasets of both the soft and the rigid modes is the same, which is 3 × 10^3^.

**Figure 4:**
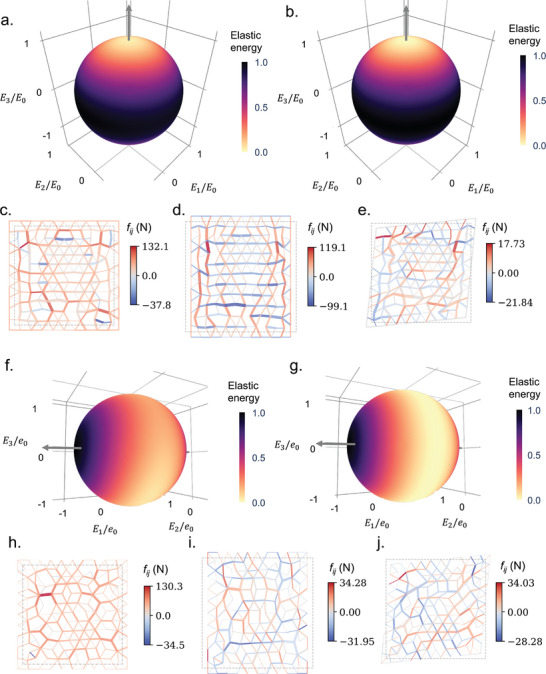
The self‐activated solid is trained to manifest uni‐mode and bi‐mode extremal materials. a,b) Elastic energy of uni‐mode solids along all directions of the strain with the magnitude being *E*_0_: a) Trained self‐activated solid; b) Homogenized ideal solid with the target stiffness tensor. c–e) Deformations of the trained uni‐mode solid, where the thickness of the lines denotes the values of the bond stiffness and the color represents the force within the active bonds: c) Hydrostatic deformation; d) Shear I deformation; e) Shear II deformation. f,g) Elastic energy of bi‐mode solids along all directions of the strain with the magnitude being *e*_0_: f) Trained self‐activated solid; g) Homogenized ideal solid with the target stiffness tensor. h–j) Deformations of the trained bi‐mode solid: h) Hydrostatic deformation; i) Shear I deformation; j) Shear II deformation. During the training, the normalized learning rate γ_0_ = 10^2^, *N*_*e*_ = 11, and the initial bond stiffness *k*_*ij*_ = 10^3^ Nm^‐1^.

Next, the two datasets are employed to train the solid to behave as a uni‐mode material. Here, we implement a lattice with 144 nodes and enforce the range of the bond stiffness as *k*_*ij*_ ∈ [10^−3^, 10^7^] Nm^‐1^. During training, we establish a series of epochs, and each of the epochs contains one hundred learning iterations followed by error validating. In each of the learning iterations, we randomly select one set of displacement and force boundary conditions from the soft or rigid mode datasets with equal possibilities. The stiffness of the bonds is updated after each of the iterations (batch size is equal to one). Once the epoch is completed, we examine the error of the stiffness tensor during the testing section. We find that the learning can be convergent after hundreds of epochs (see the figure in the Supporting Information). To evaluate the performance of the trained self‐activated solid, we calculate the elastic energy of the solid along all directions of strain with the magnitude being *E*_0_ (see **Figure** **4**a), and compare it with an ideal uni‐mode material with the effective stiffness tensor described in Equation (4) (see Figure 4b). In the figures, the elastic energy is rescaled by its maximum elastic energy, and the arrow represents the strain of the soft mode. Comparing the two figures, it can be clearly seen that even though the elastic energy of the soft mode is not exactly equal to zero, the self‐activated solid trained behaves extremely like an ideal uni‐mode material, indicating the success of training. Figure 4c –e show the trained self‐activated solid under hydrostatic, shear I, and shear II strains, respectively, where the thickness of the lines denotes the values of the bond stiffness and the color represents the force within the active bonds. Clearly, some of the bonds vanish in the lattice after training, allowing the solid to fold and realize zero modes. Further, the forces under hydrostatic and shear I deformations (rigid modes) are much greater than the forces under shear II deformation (soft mode), coinciding with the results in Figure 4a.

The learning scheme can also be applied to train the self‐activated solid to become a bi‐mode material. In a 2D space, a bi‐mode material supports only one stress state and collapses for all the other stress states. In the study, we aim to train a solid supporting the hydrostatic stress. We select a target stiffness tensor as

(5) $$\begin{array}{c} \begin{array}{c} c_{t}=\left[\begin{array}{ccc} B_{0} & 0 & 0\\ 0 & 0 & 0\\ 0 & 0 & 0 \end{array}\right] \end{array} \end{array}$$

The soft mode dataset contains linear combinations of two orthogonal soft strain states ${\left[\begin{array}{ccc} 0 & E_{2}^{\left(0\right)} & 0 \end{array}\right]}^{T}$ and ${\left[\begin{array}{ccc} 0 & 0 & E_{3}^{\left(0\right)} \end{array}\right]}^{T}$ with zero stresses, and the rigid mode dataset contains the rigid strain state ${\left[\begin{array}{ccc} E_{1}^{\left(0\right)} & 0 & 0 \end{array}\right]}^{T}$ and the corresponding rigid stress state. We then perform a learning task following the same learning scheme. Comparing Figure 4f,g, we find that the trained solid retains a rigid mode along the *E*_1_ direction as expected, even though the elastic energy of the soft modes is not zero. Further, by checking the internal forces within each of the bonds (see Figure 4h,–j), it can be seen that the forces of the two soft modes are much smaller than the forces of the rigid mode. Thus, it is safe to claim that the trained solid manifests a bi‐mode material. The success and final errors of the training here highly depend on the geometry of the lattice and the selection of the target stiffness tensor.

### 2.6 Learning Desired Time‐Varying Moduli

**Figure 5:**
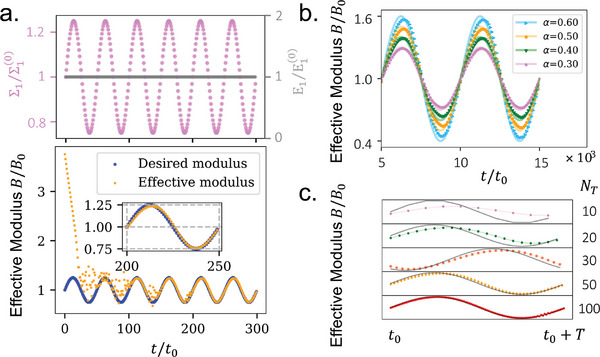
The self‐activated solid is trained with desired time‐varying moduli. a) Stress and strain states employed during training and evolution of the effective bulk modulus of the self‐activated solid. b) Evolution of the effective bulk modulus of the self‐activated solid with different target oscillation amplitudes. c) Evolution of the effective bulk modulus of the self‐activated solid with different target oscillation periods. During the training, the normalized learning rate γ_0_ = 10^2^, *N*_*e*_ = 7, and the initial bond stiffness *k*_*ij*_ = 10^5^ Nm^‐1^.

Previous sections have achieved the learning of the target stiffness tensors that are constant over time. This section explores the possibility of learning desired time‐varying moduli to facilitate the applications of time‐modulated materials.^[^ ^58^, ^59^, ^60^, ^61^, ^62^, ^63^, ^64^, ^65^ ^]^ For this purpose, we assign a sine function to the target bulk modulus *B* = *B*_0_ + α*B*_0_ *sin*(ω_0_ *t*) by varying the applied force boundary condition over time $\Sigma_{1}=\Sigma_{1}^{\left(0\right)}+\alpha \Sigma_{1}^{\left(0\right)}sin\left(\omega_{0}t\right)$ and keeping the displacement boundary condition unchanged $E_{1}=E_{1}^{\left(0\right)}$ (see the upper panel in **Figure** **5**a, where α = 0.25). In the study, we allocate 50 learning iterations within each of the oscillation periods. It can be found from Figure 5a that the trained effective bulk modulus follows the desired time‐varying modulus with negligible delays after three oscillation cycles. We also check the learning performance with different oscillation amplitudes α (see Figure 5b). When α is equal to or greater than 0.5, the learning becomes less accurate as the trained effective bulk modulus (blue points) deviates from the desired time‐varying modulus (blue curve) at the peaks and valleys of the oscillations. Further, to sustain satisfactory learning performances, the number of learning iterations *N*_*T*_ within each of the oscillation periods should be greater than 30 as indicated in Figure 5c.

## 3 Conclusion

In this work, we suggest a class of self‐activated solids and devise a local learning rule so that the solid can learn the desired stiffness tensors through repeated elastic deformations. The local learning rule is inspired by contrastive Hebbian learning, which guides the learning process by modulating the bond stiffness of the solid based on its local stain. Extensive numerical tests are performed to validate the design and learning rule. We show that the self‐activated solid can be physically trained to display the desired bulk, shear, and coupling moduli and to manifest uni‐mode and bi‐mode materials. The solid can also realize time‐varying moduli to function as time‐modulated materials. The material design and learning strategy introduced are scalable and applicable to arbitrary lattice geometries and other discrete material systems. The study could lead to new physical learning systems, beneficial to autonomous materials, machines, and robots (e.g., an adaptive robotic foot shown in the Supporting Information).

## 4 Experimental Section

### Numerical Procedures for Solving the Displacement Field of the Elastic Lattice —

The numerical approach that had been used to solve the displacement field and the internal spring forces of the elastic lattices with given displacement and force boundary conditions was illustrated in the following. Here, infinitesimal deformations was considered, and each of the springs obeys Hooke's law, which reads

(6) $$\begin{array}{c} F_{i,j}=-F_{j,i}=k_{i,j}n_{i,j}\left[n_{i,j}\cdot \left(U_{j}-U_{i}\right)\right] \end{array}$$

where

(7) $$\begin{array}{c} n_{i,j}=\frac{x_{j}-x_{i}}{\left|x_{j}-x_{i}\right|} \end{array}$$

is the unit vector along the spring direction, and **F**_*i*, *j*_ denotes the force on the *i*‐th node due to the deformation of the spring. Note that the subscript “*j*, *i*” does not denote spatial differentiation. Equilibrium then implies

(8) $$\begin{array}{c} \sum_{j}^{N}F_{i,j}+f_{i}=0 \end{array}$$

where **f**_*i*_ is the external force applied on $x_{i}^{B}$, and *N* is the number of nodes of the lattice. Using Equation (8), the displacement field of the lattice can be readily determined. However, during the calculations, the displacements on some of the nodes were given, and the reaction forces on those nodes were unknown. The number of such nodes is denoted as *M*. To solve the displacement field and the corresponding reaction forces of the lattice, Equation (8) is reformulated as

(9) $$\begin{array}{c} \sum_{j}^{N-M}k_{i,j}n_{i,j}\left[n_{i,j}\cdot \left(U_{j}-U_{i}\right)\right]=F_{i}^{B} \end{array}$$

(10) $$\begin{array}{c} \sum_{j}^{M}k_{i,j}n_{i,j}\left[n_{i,j}\cdot \left(U_{j}-U_{i}\right)\right]+f_{i}^{B}=0 \end{array}$$

(11) $$\begin{array}{c} U_{i}=U_{i}^{B},\text{for}\;i=1,2,3\text{…},M \end{array}$$

In Equations (9) ‐ (11), **U**_*i*_ and $f_{i}^{B}$ are the unknowns to be solved.

### Homogenization of the Self‐Activated Solid—

To examine the learning performance of the self‐activated solid, homogenization was performed over a trained self‐activated solid and calculate its effective stiffness tensor. An energy approach was employed in the procedure. In particular, the diagonal terms of the stiffness tensor (*B*, μ_1_, and μ_2_) were first determined by prescribing a homogenized strain state with only one nonzero component over the lattice. The homogenized strain state was applied through displacement boundary conditions. By solving the displacement field of the lattice, calculating the work done due to the reaction forces, and comparing them individually with the elastic strain energy densities of the homogenized material $E_{11}=\frac{1}{2}BE_{1}^{2}$, $E_{22}=\frac{1}{2}\mu_{1}E_{2}^{2}$, and $E_{33}=\frac{1}{2}\mu_{2}E_{3}^{2}$, effective moduli *B*, μ_1_, and μ_2_ of the stiffness tensor could be determined. Next, a homogenized strain state was prescribed with two nonzero components to determine the off‐diagonal terms of the stiffness tensor *c*_1_, *c*_2_, and *c*_3_. For example, the strain state can be ${\left[\begin{array}{ccc} E_{1} & E_{2} & 0 \end{array}\right]}^{T}$, and the homogenized elastic strain energy density becomes $E_{12}=\frac{1}{2}BE_{1}^{2}+c_{1}E_{1}E_{2}+\frac{1}{2}\mu_{1}E_{2}^{2}$. After calculating the work done due to the reaction forces (that was equal to the strain energy of the lattice), *c*_1_ can be attained using *B* and μ_1_ found from the first step. Following similar procedures, *c*_2_ and *c*_3_ can also be obtained.

## Conflict of Interest

The authors declare no conflict of interest.

## Author Contributions

Y.T. performed numerical experiments; Y.C. conceived the concept, initialized the study, and supervised the research; All authors wrote the manuscript and interpreted the results.

## Supporting information

Supporting Information

Supplemental Movie 1

## Data Availability

The data that support the findings of this study are available from the corresponding author upon reasonable request.
