# Supplementary material for: Learning Stiffness Tensors in Self‐Activated Solids via a Local Rule
Source: Adv Sci (Weinh). 2024 Mar 14;11(19):2308584. doi: 10.1002/advs.202308584 (PMC11109665; doi:10.1002/advs.202308584)
Supplement: Supplementary file 1 — Supporting Information [file ADVS-11-2308584-s001.pdf]

ADVANCED SCIENCE
Open Access

Supporting Information

for *Adv. Sci.*, DOI 10.1002/advs.202308584

Learning Stiffness Tensors in Self-Activated Solids via a Local Rule

*Yuxuan Tang, Wenjing Ye, Jingjing Jia and Yangyang Chen**

Supplementary materials for
Learning stiffness tensors in self-activated solids via a local rule

Yuxuan Tang,[1] Wenjing Ye,[1] Jingjing Jia,[2] and Yangyang Chen[1,*]

[1] *Department of Mechanical and Aerospace Engineering, The Hong Kong University of Science and Technology, Clear Water Bay, Kowloon, Hong Kong*

[2] *Institute of Materials Engineering, Beijing Institute of Collaborative Innovation, Beijing, 100094 China*

S1. REALIZATION OF THE ACTIVE BOND WITH TUNABLE STIFFNESS

To realize an elastic bond with tunable stiffness, we suggest a mechanical structure consisting of two springs and a regulator driven by a stepper motor (see Fig. S1a). In the design, one of the springs (the green spring in Fig. S1a) connects the two ends of the structure, while the other spring (the brown spring in Fig. S1a) connects only one end of the structure and the other end of the spring is free. The regulator between the two springs is rigid, which separates each of the two springs into two parts. The vertical position of the regulator is tuned by a stepper motor located at the lower end of the structure (yellow cylinder in Fig. S1a) through a grooved shaft (green cylinder in Fig. S1a). When the regulator moves, the lengths of the two springs below and above the regulator vary, giving rise to a change in the effective stiffness of the whole structure.

To quantify the effective stiffness, we model the mechanical structure using three springs as shown in Fig. S1b. Springs I and II in the figure denote the spring segments below the regulator in Fig. S1a, and spring III represents the

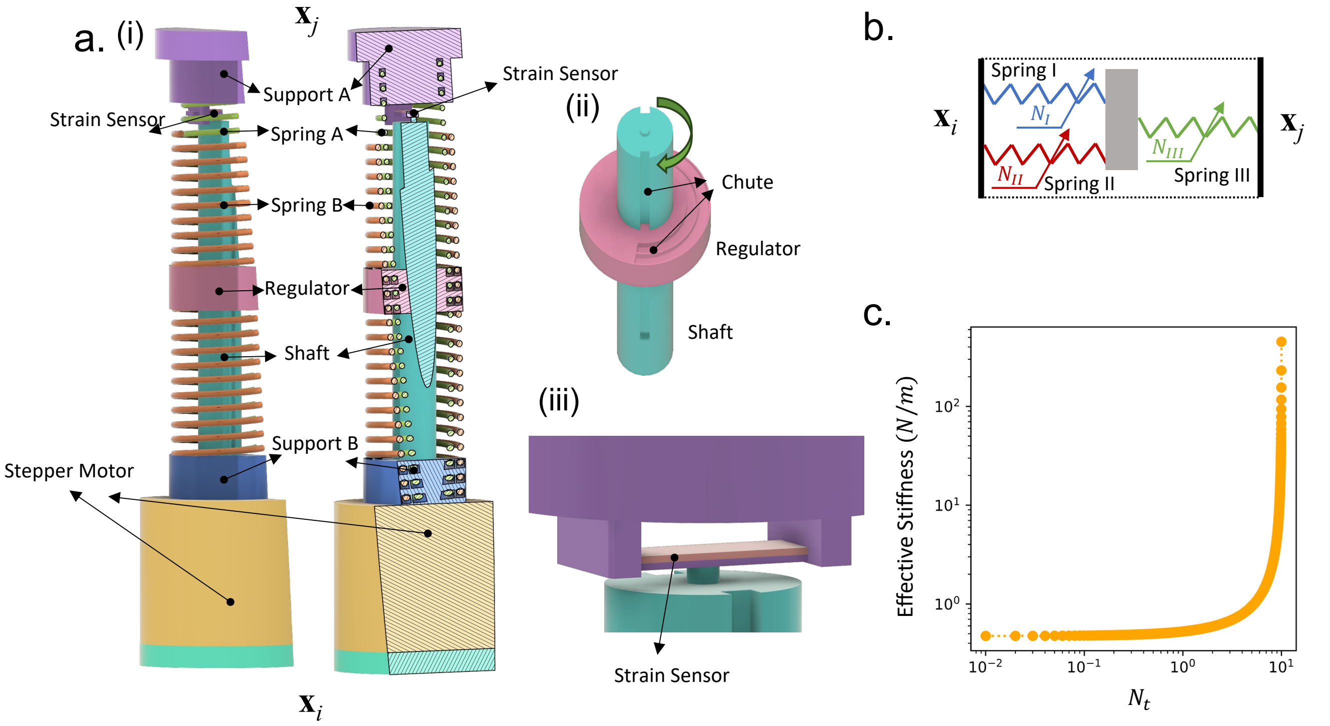

Fig. S1. Realization of the active bond with tunable stiffness. (a) Design of a mechanical structure that consists of two springs and a regulator driven by a step motor to realize the active bond with tunable stiffness. (b) Lumped spring model of the design of the active bond. (c) Effective stiffness of the active bond with different numbers of windings of the springs below the regulator.

* maeychen@ust.hk

spring segment above the regulator. The effective stiffness of the spring system then reads $K_e = \frac{(K_1+K_2)\cdot K_3}{K_1+K_2+K_3}$, where K_1, K_2 and K_3 are the stiffness of springs I, II, and III, respectively. In practice, the stiffness of the individual spring can be calculated by $K_i = \frac{G_i d_i^4}{8D_i^3 N_i}$, where G_i, d_i, D_i, and N_i represent the shear modulus, the wire diameter, the inner spring diameter, and the number of windings of the i-th spring, respectively. When the regulator moves vertically, the numbers of windings of the three springs alter, and the stiffness of springs I, II, and III, as well as the effective stiffness of the spring system, vary. As an illustrative example, we examine the tuning ability of the design in Fig. S1c, where N_t denotes the number of windings of the springs below the regulator. In the example, we select $d_A = 0.2$ mm, $G_A = 80$ GPa, $D_A = 1.5$ cm, $N_A = 10$ for the green spring, and $d_B = 3$ mm, $G_B = 80$ GPa, $D_B = 3$ mm, $N_B = 10$ for the brown spring. It can be seen from the figure that the effective stiffness can vary from 0.5 N/m to around 400 N/m as the N_t changes from 0 to 10. The tuning range is enough for most of the examples demonstrated in the main text.

To measure the strain of the system, learnabilitywe mount a small elastic beam attached with a piezoelectric sensor between the free end of the grooved shaft and the upper support of the system. In the design, the bending stiffness can be negligible compared to the stiffness of the spring system.

S2. LEARN DESIRED SHEAR MODULI μ_1 AND μ_2

Following a similar learning procedure as demonstrated in the main text for the bulk modulus, here we show the numerical testing results regarding the learning of shear moduli μ_1 and μ_2. We first examine the effects of lattice geometries on the success of learning. Figs. S2a and S2e illustrate the evolution of the force error for learning the desired μ_1 and μ_2, respectively, with lattices containing 58, 98, and 144 nodes. Each of the three curves in the figures contains the simulation results with ten different lattices. All other parameters are the same as those used in Fig. 2d in the main text. The results in the two figures show that the force error becomes negligibly small at the end of each test, indicating the success of the learning tasks for μ_1 and μ_2. This point is further illustrated in Figs. S2b and S2f for μ_1 and μ_2, respectively. During simulations, we implement different initial bond stiffness for the lattice, as we did in Fig. 2e in the main text. As expected, in Fig. S2b, μ_1 reaches the desired value, while $c_1 = c_3 = 0$, and, in Fig. S2f, μ_2 reaches the desired value, while $c_2 = c_3 = 0$. Additionally, the uncontrolled parameters are randomly distributed. Similar to Fig. 2f in the main text, the upper bound of the bond stiffness also enforces a bound on the maximum achievable shear moduli μ_1 and μ_2. Figs. S2c and S2g show the force error in the final training step with different target shear moduli μ_1 and μ_2, respectively. As shown in the figures, lower target shear moduli can lead to smaller final errors. When the target shear moduli reach a critical value, the error increases sharply, indicating the bound of the maximum achievable shear moduli. A similar linear relationship between the upper bound of the bond stiffness and the bound on the maximum achievable shear moduli is observed. Finally, we examine the effects of learning rates for different target shear moduli μ_1 and μ_2 in Figs. S2d and S2h, respectively, where optimal learning rates can be identified for different learning targets.

S3. EVOLUTION OF ERRORS DURING THE TRAINING OF EXTREMAL MATERIALS

To train extremal materials, we organize the training processes into a series of epochs. Each epoch consists of one hundred learning iterations followed by modulus testing. During testing, we calculate the effective stiffness tensor of the self-activated solid and compare it with the desired tensor with the error defined by $e_r^m = \sum_{ij} \| \frac{\mathbf{C}_{ij} - \mathbf{C}_{ij}^{\texttt{desired}}}{B_0} \|$ with $\mathbf{C}_{ij}$ and $\mathbf{C}_{ij}^{\texttt{desired}}$ denoting the effective and desired stiffness tensors, respectively. Figs. S3a and S3b show the evolution of the errors during the training of uni-mode and bi-mode materials, respectively. It can be clearly seen that the errors reduce rapidly and converge to a small value in just a few hundred learning epochs.

S4. AN APPLICATION OF THE SELF-ACTIVATED SOLID

We suggest implementing the self-activated solid proposed in this study into the application of adaptive robotic feet (see Fig. S4a). In particular, this adaptive robotic foot can accommodate ground surfaces of any shapes and remain flat and the same height on the top surface after the training for accurate and stable operations of sensitive optical and electrical devices. To validate the feasibility, we perform a series of numerical simulations with different ground surfaces. During the training, the force-prescribed state is formed by the payload of the robotic foot. To be simple, we apply a vertical force F_r on each of the points on the top surface of the self-activated solid. The displacement-prescribed state is realized by applying a vertical displacement V_r on each of the points on the top surface. Before the

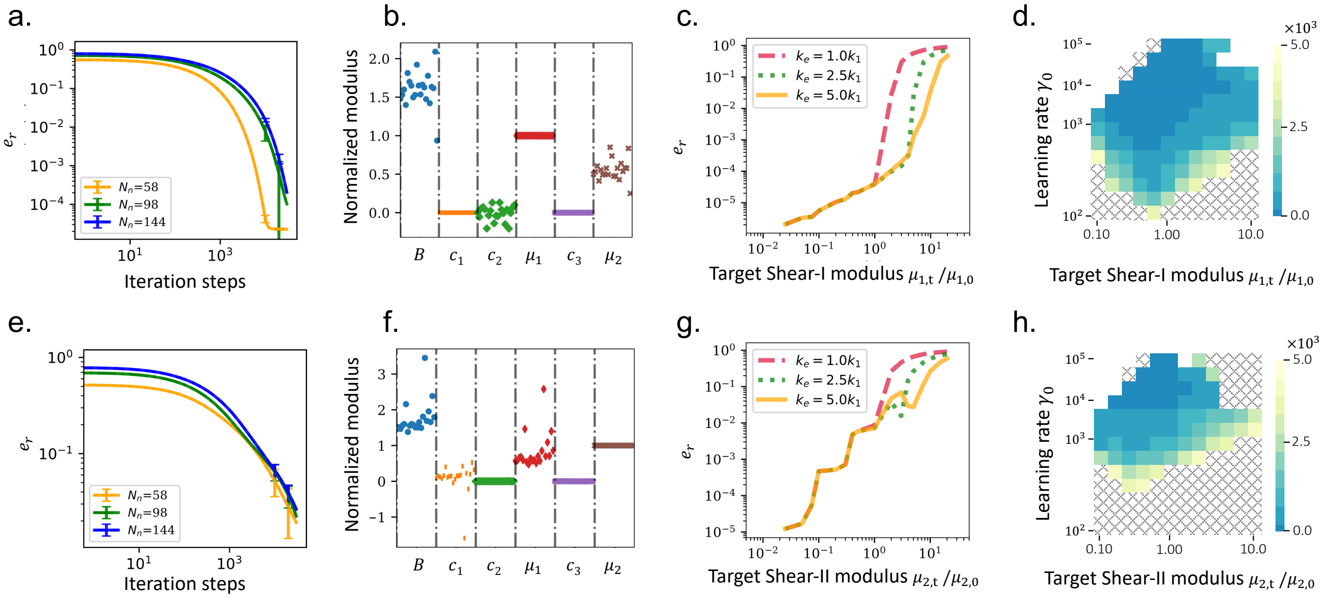

Fig. S2. Learn desired shear moduli μ_1 and μ_2. (a) Evolution of the force error of the self-activated lattice with different numbers of nodes during learning. (b) Normalized effective shear I moduli of the trained self-activated solid with different initial bond stiffness. (c) Final force error of the trained self-activated solid with different target shear I moduli, where different upper bounds on the bond stiffness are implemented. (d) Numbers of learning steps needed for convergence with different target shear I moduli and learning rates. (e) Evolution of the force error of the self-activated lattice with different numbers of nodes during learning. (f) Normalized effective shear II moduli of the trained self-activated solid with different initial bond stiffness. (g) Final force error of the trained self-activated solid with different target shear II moduli, where different upper bounds on the bond stiffness are implemented. (h) Numbers of learning steps needed for convergence with different target shear II moduli and learning rates.

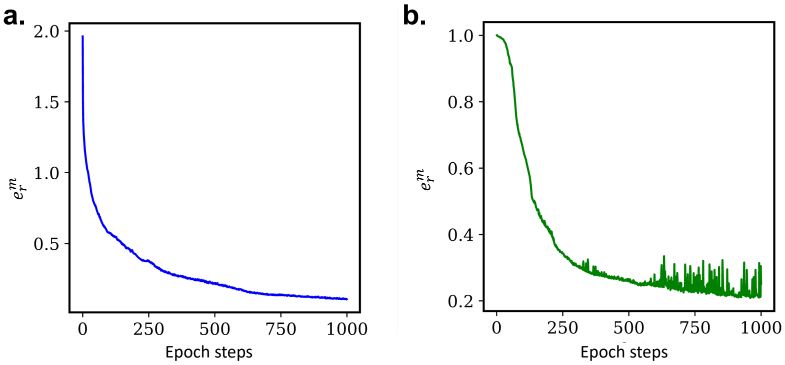

Fig. S3. Evolution of errors during the training of extremal materials. (a) Uni-mode material; (b) Bi-mode material.

training, we initialize the self-activated solid according to Fig. 2a. Fig. S4b shows the self-activated solids after the training with different ground surfaces. In the figure, the widths of the lines denote the values of the bond stiffness after the training. Clearly, the top surface, as expected, can remain flat and the same height for ground surfaces with different shapes after the training. Good accuracy can be obtained after a few thousand of learning steps (see Fig. S4c).

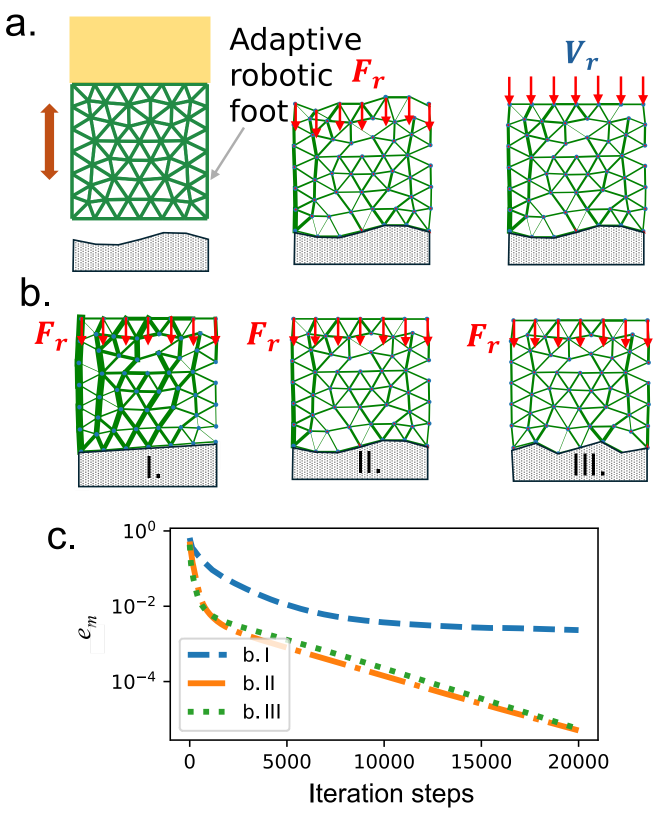

Fig. S4. (a) An adaptive robotic foot designed using the self-activated solid that can accommodate ground surfaces of any shapes and remain flat and the same height on the top surface after the training. (b) The self-activated solids after the training with different ground surfaces. (c) The relative error in different iteration steps.
